# Rational Discovery of (+) *(S)* Abscisic Acid as a Potential Antifungal Agent: a Repurposing Approach

**DOI:** 10.1038/s41598-018-26998-x

**Published:** 2018-06-04

**Authors:** Mohammed A. Khedr, Alberto Massarotti, Maged E. Mohamed

**Affiliations:** 1 College of Clinical Pharmacy, King Faisal University, P.O. 400, Al-Hasaa, 31982 Saudi Arabia; 2 Department of Pharmaceutical Chemistry, Faculty of Pharmacy, Helwan University, Ein Helwan, Cairo, 11795 Egypt; 3 Dipartimento di Scienze del Farmaco, Università del Piemonte Orientale “A. Avogadro”, Largo Donegani 2, 28100 Novara, Italy; 4 Department of Pharmacognosy, Faculty of Pharmacy, University of Zagazig, Zagazig, 44519 Egypt

## Abstract

Fungal infections are spreading widely worldwide, and the types of treatment are limited due to the lack of diverse therapeutic agents and their associated side effects and toxicity. The discovery of new antifungal classes is vital and critical. We discovered the antifungal activity of abscisic acid through a rational drug design methodology that included the building of homology models for fungal chorismate mutases and a pharmacophore model derived from a transition state inhibitor. Ligand-based virtual screening resulted in some hits that were filtered using molecular docking and molecular dynamic simulations studies. Both *in silico* methods and *in vitro* antifungal assays were used as tools to select and validate the abscisic acid repurposing. Abscisic acid inhibition assays confirmed the inhibitory effect of abscisic acid on chorismate mutase through the inhibition of phenylpyruvate production. The repositioning of abscisic acid, the well-known and naturally occurring plant growth regulator, as a potential antifungal agent because of its suggested action as an inhibitor to several fungal chorismate mutases was the main result of this work.

## Introduction

**Figure 1:**
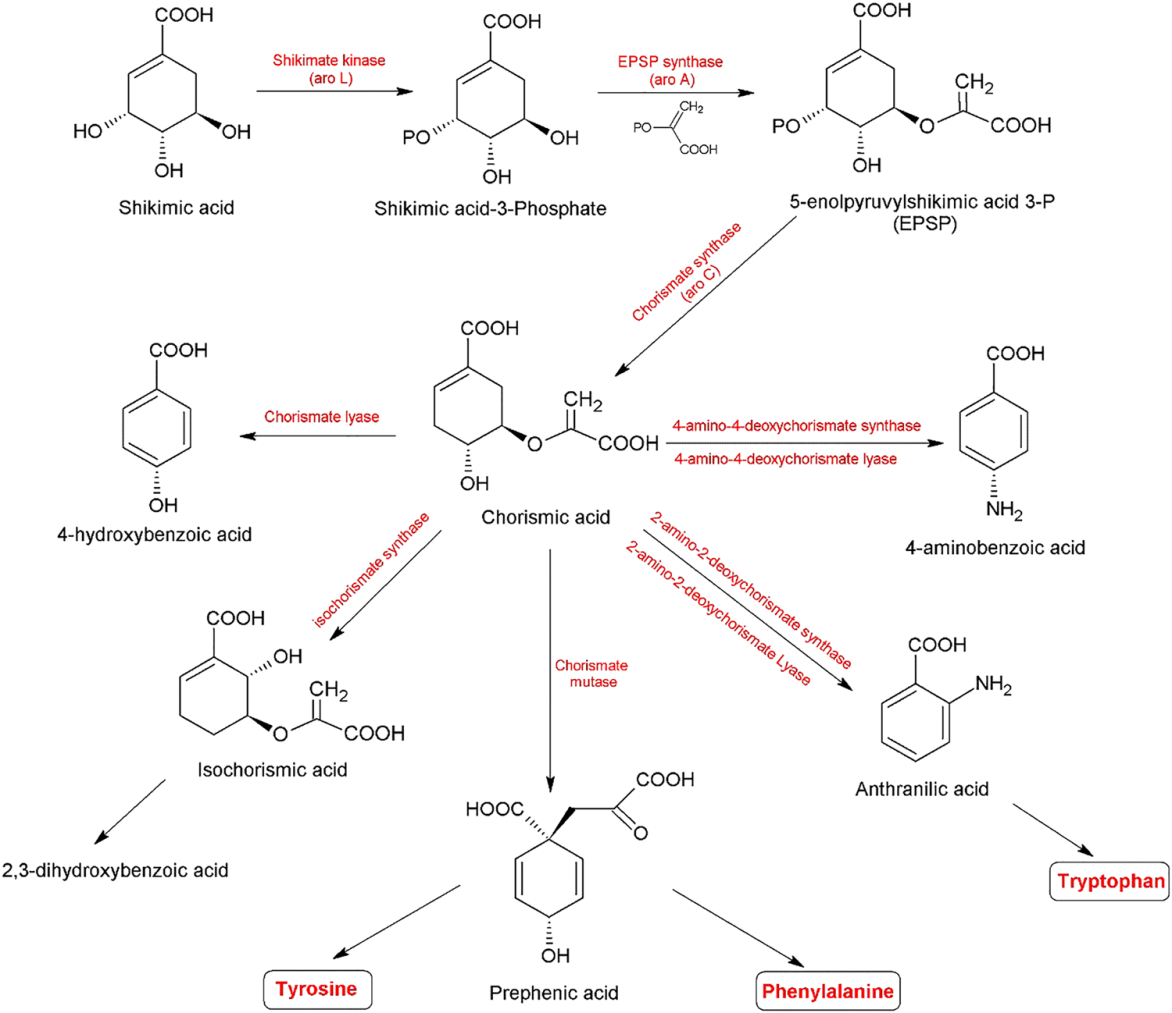
Shikimic acid biosynthetic pathway as a precursor for essential amino acids in plants, fungi and bacteria.

The shikimate pathway provides one of the main channels for the biosynthesis of aromatic compounds, primarily the aromatic amino acids phenylalanine, tyrosine, and tryptophan, in plants and microorganisms (Fig. 1). The pathway converts phosphoenolpyruvate and erythrose 4-phosphate to chorismate through seven enzymatically catalysed steps. Chorismate serves as a precursor for the synthesis of a variety of aromatic compounds, such as p-aminobenzoic, 2,3-dihydroxybenzoic, prephenic and anthranilic acids (Fig. 1)^1–3^.

This pathway was originally discovered in plants. However, it is now established that several essential amino acids are biosynthesized in many organisms such as bacteria^4,5^, *Mycobacterium tuberculosis*^6^, fungi, and parasites^7^ via such a pathway. The absence of this pathway in animals, which obtain these essential amino acids through their diet, makes the enzymes of this pathway ideal targets for the development of new antimicrobial agents and nonhazardous herbicides^8^. For example, glyphosate, in addition to its wide use as a commercial herbicide, is an effective anti-microbial and anti-parasitic drug that inhibits 5-enolpyruvyl shikimate-3-phosphate (EPSP) synthase^7,9^.

Chorismate is a central metabolite in the shikimate pathway and a branch point for at least five different metabolic pathways in microorganisms (Fig. 1)^4^. The aromatic amino acids L-phenylalanine and L-tyrosine are formed from chorismate via prephenate, which undergoes either decarboxylation/dehydration or decarboxylation/dehydrogenation, followed by transamination to form the corresponding amino acids (Fig. 1). Chorismate mutase/prephenate dehydrogenase is a bifunctional enzyme that occurs as a homodimer with a molecular weight of approximately 78,000 in *Escherichia coli*, and it is involved in phenylalanine and tyrosine biosynthesis in bacteria and fungi^4,10,11^. To summarize, the absence of this type of enzyme in mammals, including humans, makes it a promising target for the development of new anti-mycobacterial, antifungal and antibacterial drugs^12,13^.

Molecular modelling tools have recently been discovered and developed for use in the design, modification and discovery of new chemical entities. These tools have been used to predict the biological activities of unknown compounds compared to reference drugs. Recent advances in computer-aided drug design have enabled scientists to interpret the mode of action of many enzyme inhibitors. The use of such tools in the process of drug repurposing may be of great interest, providing a powerful way to find new medical uses either for old drugs or for those with nonmedical use in new dosage forms. Drug discovery tools help to identify new targets of known drugs in new diseases^14^. Rational drug design is defined as the development of small molecules with desired properties for targets, biomolecules (proteins or nucleic acids), whose functional roles in cellular processes and 3D structural information are known^15^. It is one of the commonly used tools for drug discovery, and it has been used widely in pharmaceutical industries.

The main aim of this work was to discover a novel chorismate mutase inhibitor as a potential antifungal agent using ligand-based virtual screening, homology modelling, molecular docking and molecular dynamic simulations as a rational approach. The results of the *in silico* studies were supported by *in vitro* antifungal and enzyme inhibition assay investigations.

## Results

The design of inhibitors based on structural information derived from chorismate mutase enzymes, which are present in microorganisms (archaebacteria, eubacteria, and yeast), fungi, and plants but not in animals and humans, provides the potential for the discovery of new selective antifungal agents. Unfortunately, the crystal structures of the chorismate mutases for most fungal strains, such as *Candida albicans*, *Candida parapsilosis*, *Aspergillus niger*, *Trychophyton rubrum* and *Trychophyton mentagrophytes*, are not available. In addition, the absence of any data related to either FDA-approved drugs or novel lead compounds that target the chorismate mutase enzyme makes the process more difficult to find at least one potential compound for further development.

### Establishment of homology models for chorismate mutase in different fungal strains

To search for chorismate mutase inhibitors that could be used as potential antifungal drugs, the first step was to build the homology model for chorismate mutases of *C. albicans*, *C. parapsilosis*, *A. niger*, *T. rubrum* and *T. mentagrophytes* strains, which represent different types of fungal species. The FASTA sequences for the previous fungal strains were downloaded from the UniPort protein data bank. However, the sequence for *T. mentagrophytes* could not be retrieved. A sequence similarity search was performed using MOE 2014.09 software to determine the best template with the highest identity to be used for building the homology models, and the crystal structure of *Saccharomyces cerevisiae* was acquired as the most suitable template (Table 1).

**Table 1:** Percentage of identity of different fungal chorismate mutase protein in respect to *S. cerevisiae* chorismate mutase.

Fungal Strain	UniProt Code	Percentage of identity	Number of residues
*C. albicans*	Q59TS4	62.45%	268
*C. prapsilosis*	G8BD21	58.36%	267
*T. rubrum*	F2SCL7	36.778%	325
*A. niger*	A2R3Z4	44.00%	266
*S. cerevisiae*	P32178	—	256

These results were obtained using a sequence similarity search using MOE 2014.09 software.

Homology models for *C. albicans*, *C. parapsilosis*, *A. niger* and *T. rubrum* were constructed using MOE 2014.09 software. The resulting models were validated by computing the root-mean-square deviation (RMSD) from the template and analysis of the Ramachandran plot results for each model (Supplementary Data).

### Determination of the active substrate-binding site

**Figure 2:**
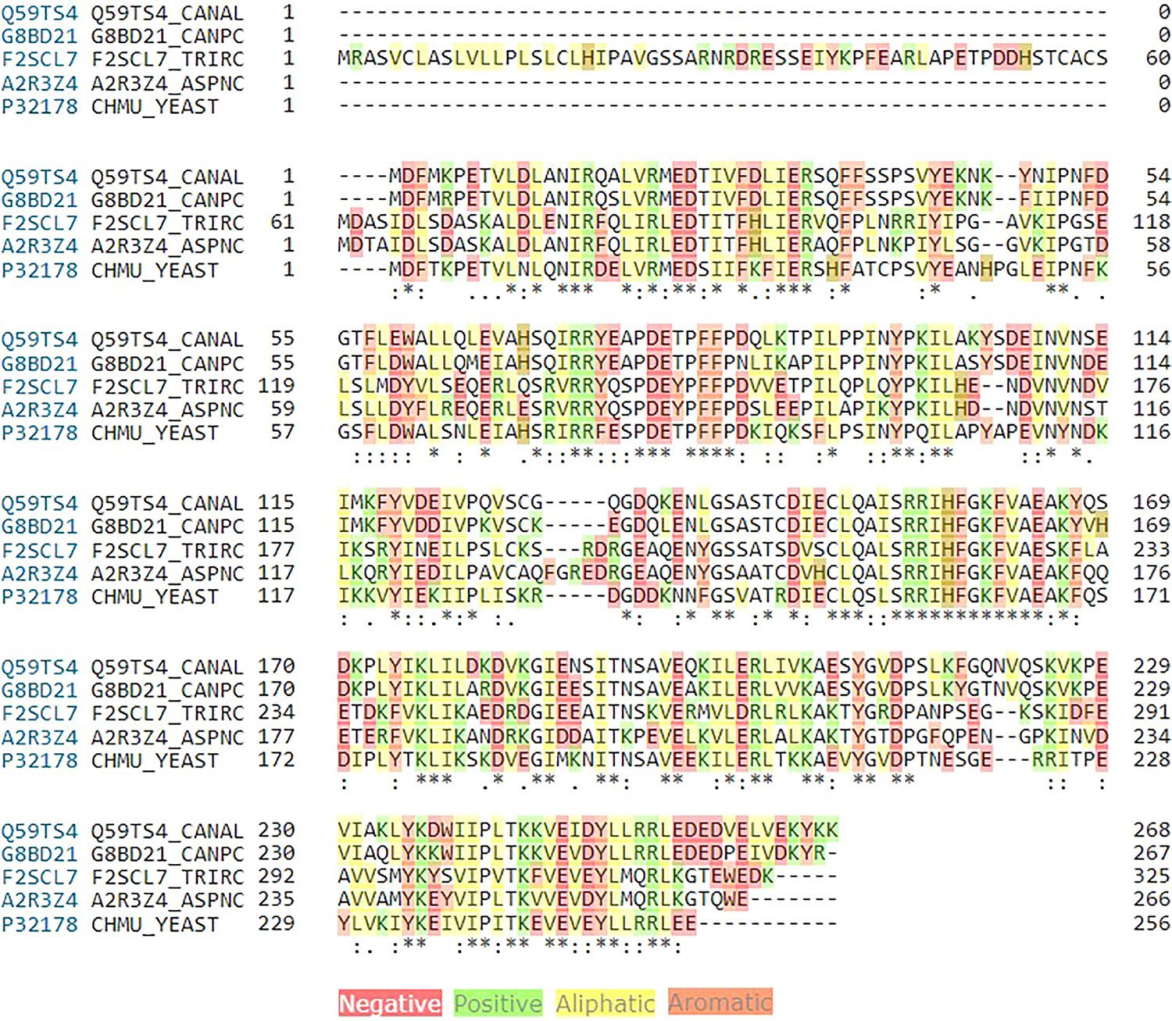
Protein sequence alignment for chorismate mustase proteins of different fungi strains, showing different identical sites. This sequence alignment was done using Clustal omega software (http://www.ebi.ac.uk/Tools/msa/clustalo/). The fungal stains under investigation are *C. albicans* (Q59T54), *C. prapsilosis* (G88D21), *T. rubrum* (F2SCL7), *A. niger* (A2R3Z4), *S. cerevisiae* (P32178).

To determine the active substrate-binding site, protein-sequence alignment for different chorismate mutase enzymes of the previous fungi strains was performed. The results (Fig. 2) identified conservative regions in all sequences (155-SRRIHFGKFVAE-166) that should be required for the enzyme activity and represent the active site of substrate binding.

### Design of a pharmacophore model for fungal chorismate mutase inhibitors

**Figure 3:**
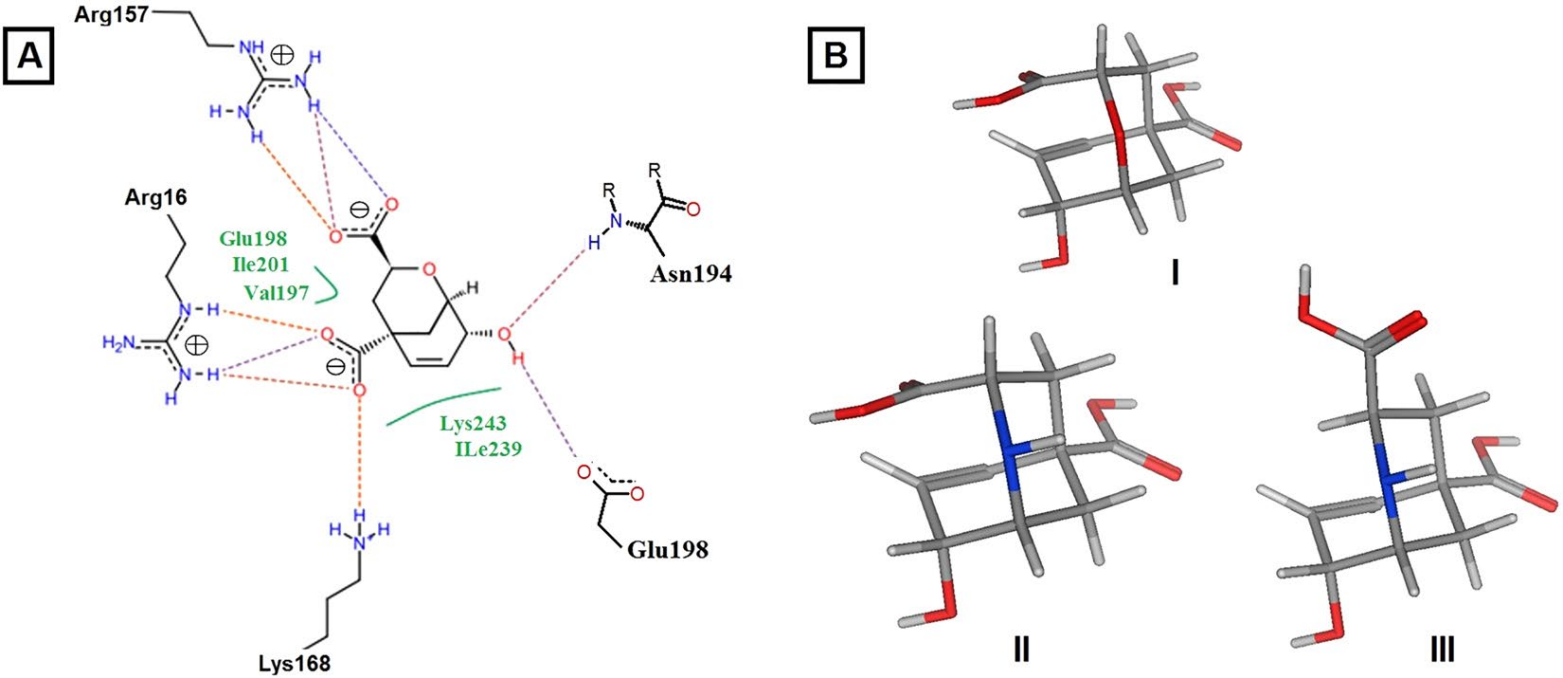
(**A**) The possible binding mode of *endo*-oxabicyclic inhibitor inside the *S. cerevisiae* chorismate mutase. (**B**) Chemical structures of different transition state analogue inhibitors.

Previous studies sought an explanation of the mechanism of chorismate mutase enzyme inhibition. In these studies, they found that *endo*-oxabicyclic and aza-bicyclic transition state inhibitors were the most effective simulators for the enzyme. In addition, these inhibitors provide good insights into the requirements for an ideal inhibitor. However, these *endo*-oxabicyclic and aza-bicyclic inhibitors were not further developed, possibly due to the difficulty of chemical synthesis. Our aim was to derive the pharmacophoric features of these inhibitors (ligand-based approach) by building a pharmacophore model to be used in a virtual screening process. With the aim of designing a ligand-based pharmacophore model for the discovery of novel fungal chorismate mutase inhibitors, the crystal structure of yeast chorismate mutase in complex with an *endo*-oxabicyclic transition-state analogue inhibitor^16^ was studied. The complex provided very important insights into the inhibitor’s binding mode to the enzyme. The same *endo*-oxabicyclic transition-state analogue inhibitor was also reported in complex with different chorismate mutases for both *Bacillus subtilis*^17^ and *E. coli*^18^, indicating that this inhibitor has all the pharmacophores required for the inhibition of such enzymes (Fig. 3A). In addition, other transition-state analogue inhibitors were discovered for yeast chorismate mutase (Fig. 3B). However, they share similar structural features as *endo*-oxabicyclic.

**Figure 4:**
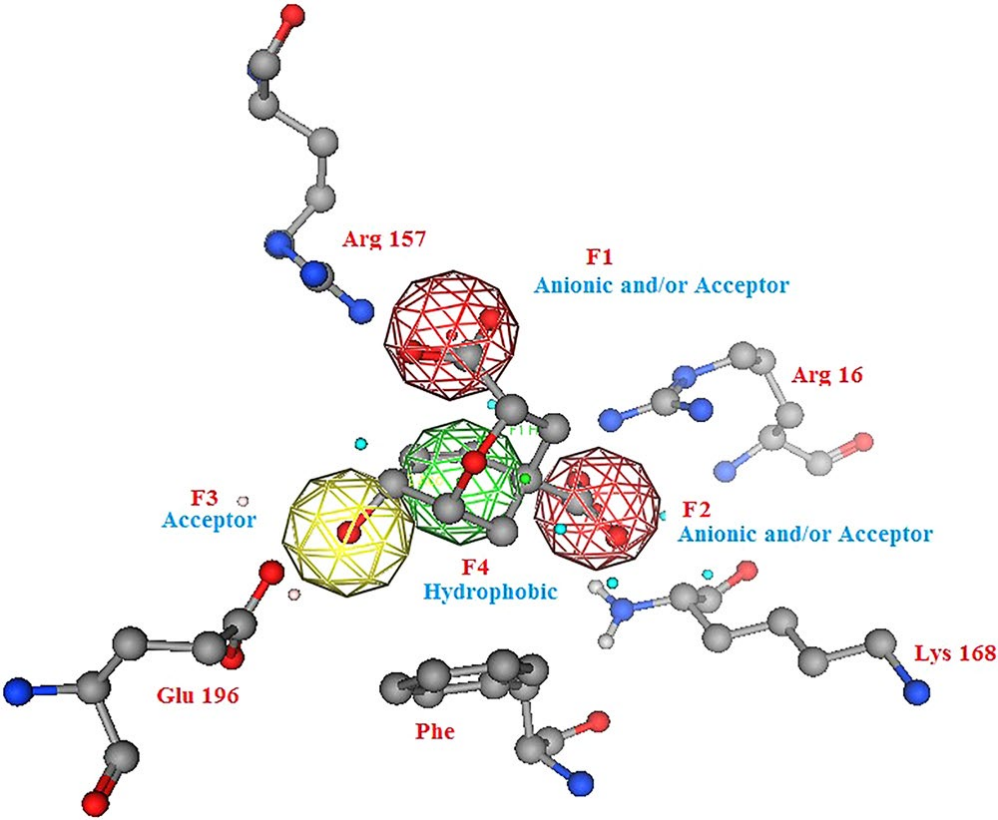
A Pharmacophore model for fungal chorismate mutase inhibitors. The model was designed after studying the crystal structure of yeast chorismate mutase in complex with endo-oxabicyclic transition-state analogue inhibitor^16^. The established model composed of four pharmacophoric features.

The analysis of the *endo*-oxabicyclic and aza-bicyclic inhibitors mode of binding revealed that the two anionic and/or acceptor pharmacophoric features of the two carboxylate groups were essential in supporting the salt bridge formation with Arg157, Arg16 and Lys168. The hydroxyl group acts as an acceptor with its oxygen atom to form a strong hydrogen bond with Asn194 and as a hydrogen donor to form another hydrogen bond with Glu198 (Fig. 3A). These data were retrieved from the inactive form of the yeast chorismate mutase bound to the Tyr amino acid allosteric inhibitor. Neither the oxygen atom nor the nitrogen atom in the bicyclic system of inhibitors was involved in any binding. In addition, the six-membered cyclohexene ring was the most common in the inhibition of the chorismate mutases of *B. subtilis*, *E. coli* and *S. cerevisiae*^19^. The *endo*-oxabicyclic inhibitor has 0.007 µM inhibitory activity on chorismate mutase. Currently, there are no inhibitors for fungal chorismate mutases. Therefore, data from the yeast inhibitors were used to build a pharmacophore model for the virtual screening process. The established model comprised four pharmacophoric features, as shown in Fig. 4.

The pharmacophore query model was used to screen of a number of databases, such as the MOE 2014.09 and ZINC databases. The screening involved 160,000 lead-like compounds, and a conformational import was conducted to derive all possible conformations of the compounds screened. The selection in the final output was filtered according to Lipinski’s rule of five for drug-like properties. As a result, 25 compounds were selected as top-ranked hits with an RMSD < 1.00 and retention of all pharmacophore features. According to the RMSD values resulting from the virtual screening process, hits **1, 2, 4, 5, 7, 9, 10, 12, 13, 16**, and **20** showed the lowest RMSD values (Supplementary Material: Fig. SI).

### Docking of potential fungal chorismate mutase inhibitors

Molecular docking of the top-ranked hits with low RMSD values was performed inside the transition-state binding site of the four chorismate mutase homology models of *C. albicans*, *C. parapsilosis*, *A. niger* and *T. rubrum*. The RMSD values of the hits and docking results are summarized in Table 2. According to the docking results, hits with one C=C bond in the cyclohexene ring showed higher docking scores than those with two C=C bonds. In addition, hits with pentanoic acid side chains such as compound (**13**) and methylpent-2-enoic acid such as compounds (**9**) and (**10**) showed higher free binding energies and low clash scores than propionic acid side chains. Compound (**13**) showed the highest docking sore (−38.91 kcal/mol) and a relatively low (4.15) clash score. Unfortunately, compound (**13**) is not available commercially. The second two compounds in ranking were (**9**) and (**10**), with free binding energy scores of (−31.65 kcal/mol) and (−31.45 kcal/mol), respectively. They showed exactly the same binding mode that was similar to the transition-state analogue inhibitor. Similarly, neither compound is available commercially because they both have two chiral centres that may result in four isomers. The presence of several isomers could affect the biological and pharmacological activities of these compounds and may result in severe side effects due to the toxicity of one of the isomers.

**Table 2:** Docking results of the top-ranked 11 compounds resulted from database screening by the established pharmacophore model for fungal chorismate mutase inhibitors.

Compounds	RMSD (Ǻ)	*C. albicans*		*C. parapsilosis*		*A. niger*		*T. rubrum*	
		ΔG (Kcal/mol)	Clash score	ΔG (Kcal/mol)	Clash score	ΔG (Kcal/mol)	Clash score	ΔG (Kcal/mol)	Clash score
Compound 1	0.95	−27.18	5.75	−28.13	5.30	−26.93	4.62	−24.91	5.52
Compound 2	0.86	−31.46	6.11	−30.45	4.56	−28.17	3.36	−26.99	3.44
Compound 4	0.82	−29.19	5.85	−19.11	4.82	−29.15	3.65	−26.35	3.49
Compound 5	0.98	−21.35	7.24	−19.89	5.60	−27.56	4.84	−23.68	3.30
Compound 7	0.96	−28.73	6.95	−20.05	4.90	−26.95	5.28	−27.00	4.08
Compound 9	0.72	−31.65	4.28	−30.85	4.18	−30.89	3.25	−28.45	3.85
Compound 10	0.75	−31.45	4.30	−31.11	4.56	−31.02	3.33	−28.70	3.75
Compound 12	0.84	−19.85	8.40	−20.85	4.25	−19.85	4.51	−23.27	4.73
Compound 13	0.65	−38.91	4.15	−35.14	4.45	−31.55	3.46	−29.08	3.81
Compound16	1.01	−16.85	5.21	−17.65	4.45	−15.65	4.25	−25.68	5.60
Compound 20	1.00	−14.97	9.80	−17.19	6.08	−19.86	4.51	−18.20	4.85
*Endo*-oxabicyclic transition-state analogue		−40.52	2.59	−38.31	2.54	−31.53	3.92	−33.16	4.87

Docking was done by Leadit 2.1.8. The free energy of binding is represented by ΔG (Kcal/mol).

A structure-similarity search based on compounds (**9**) and (**10**) using both SciFinder and PubChem midlines was performed to obtain a highly similar compound that could be easily synthesized and/or purchased. Both searches resulted in one common compound with 98% similarity: abscisic acid.

### Stereoisomers of abscisic acid

**Figure 5:**
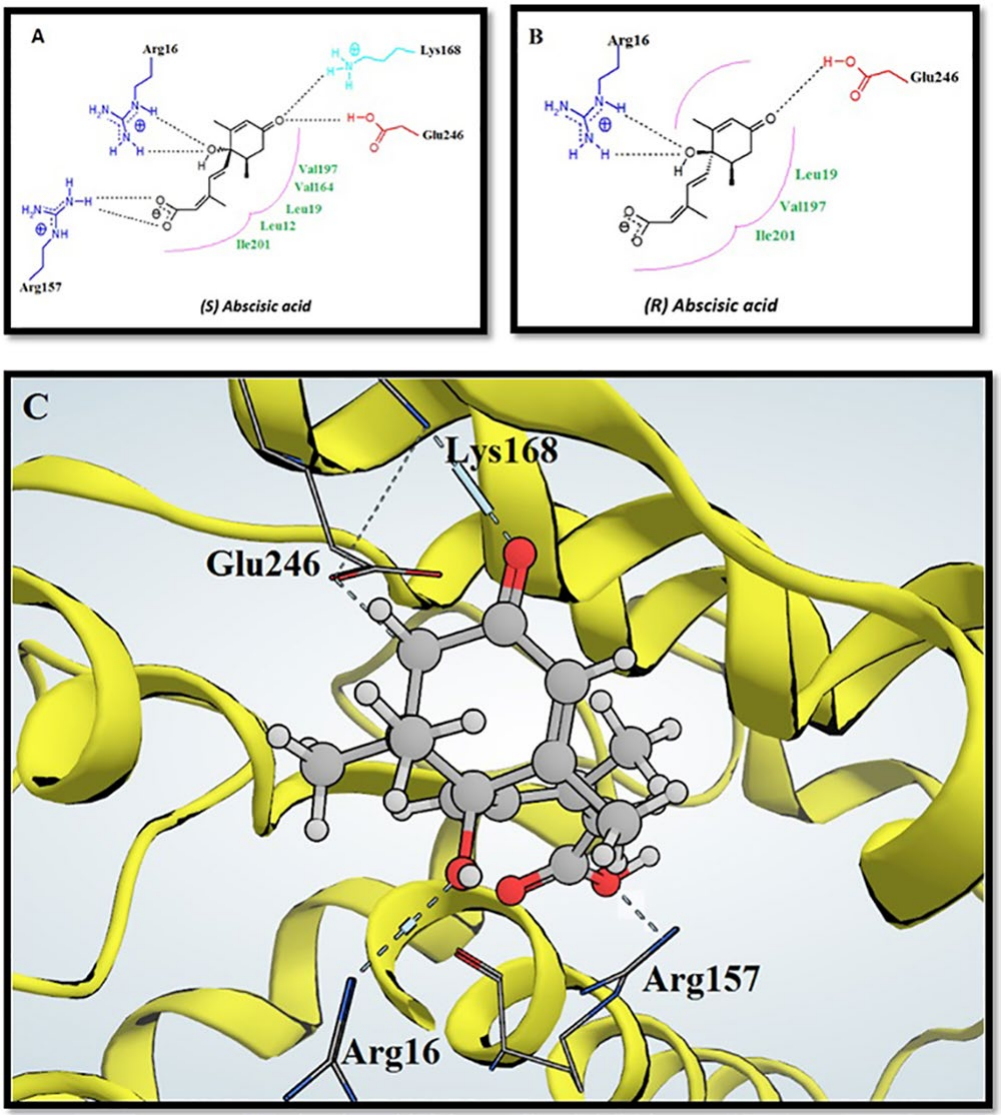
(**A**) Best binding mode of (*S*) Abscisic acid in *C. albicans* chorismate mutase substrate binding site. (**B**) Best binding mode of (*R*) Abscisic acid in *C. albicans* chorismate mutase substrate binding site. (**C**) The 3D binding mode of (*S*) Abscisic acid in *C. albicans* chorismate mutase.

ABA has a 5-(1-hydroxy-2,6,6-trimethyl-4-oxo-2-cyclohexene-1-yl)-3-methyl-pentadienoic acid scaffold, which may be in (*S*) or (*R*) stereo-configuration, and both isomers are commercially available (Supplementary Material: Fig. SII). To verify the activity of abscisic acid as a fungal chorismate mutase inhibitor and to identify which of its two isomers have the best activity, a molecular docking study was performed for both abscisic acid isomers (Fig. 5) against the four homology models and compared to the docking of *endo*-oxabicyclic transition-state analogue inhibitors.

**Figure 6:**
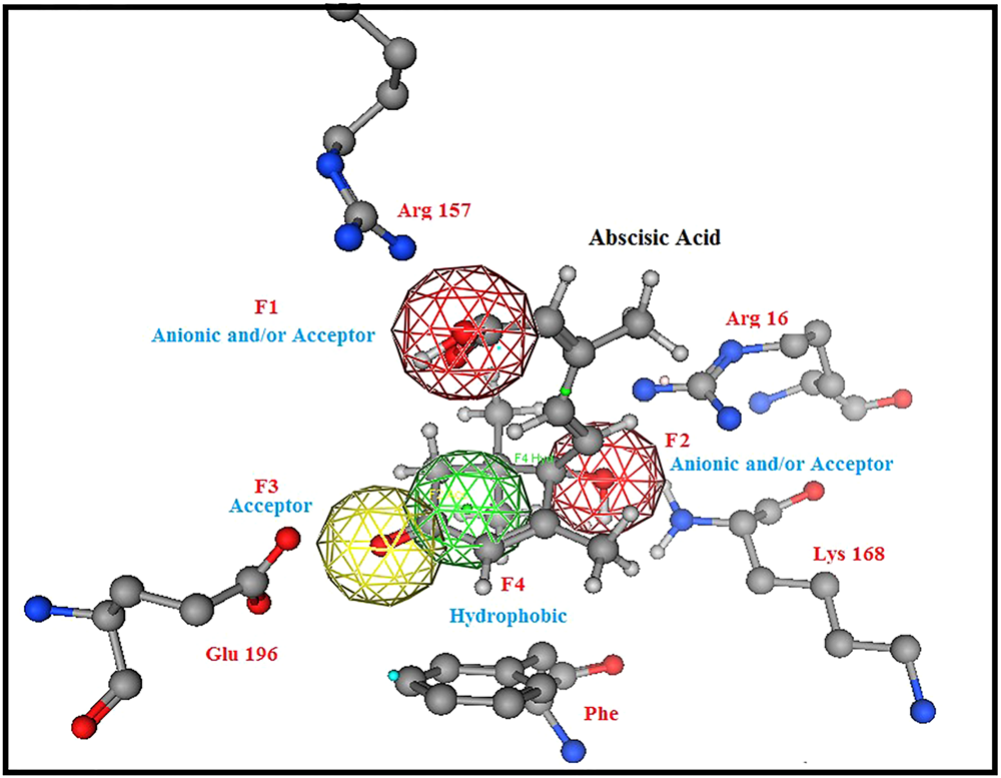
One of the generated conformations for ABA (*S*) isomer, matched with the established pharmacophore model for fungal chorismate mutase inhibitors.

Table 3 shows the docking results that indicate that the (*S*) configuration was better than the (*R*) configuration in terms of the binding mode, free energy binding score and lowest clash, which resembles those of the *endo-*oxabicyclic transition state-analogue inhibitor. Figure 6 shows one of the generated conformations for the abscisic acid (*S*) isomer, which was matched with the previously established pharmacophore model for fungal chorismate mutase inhibitors.

**Table 3:** Docking results of Abscisic acid isomers using Leadit 2.1.8. The free energy of binding is represented by ΔG (Kcal/mol).

	ΔG Free binding energy kcal/mol	Lipophilic contribution score	Clash score	Ligand entropy conformation score
***C. albicans*** **chorismate mutase**				
(*S*) configuration	−32.22	−7.89	4.24	2.80
(*R*) configuration	−28.84	−8.09	6.11	2.80
***C. parapsilosis chorismate mutase***				
(*S*) configuration	−32.11	−7.89	4.20	2.80
(*R*) configuration	−29.89	−8.09	5.95	2.80
***A.niger chorismate mutase***				
(*S*) configuration	−32.25	−7.89	4.22	2.80
(*R*) configuration	−28.54	−8.09	6.14	2.80
***T. rubrum chorismate mutase***				
(*S*) configuration	−35.45	−7.89	4.22	2.80
(*R*) configuration	−27.80	−8.09	5.60	2.80

The best configuration of the (*S*) configuration was fixed in each homology model of *C. albicans*, *C. parapsilosis*, *A. niger* and *T. rubrum* after the docking process. Then, the four chorismate mutase-ligand complexes were subjected to a molecular dynamic simulation to test the stability of (+) (*S*) abscisic acid (ABA) binding inside the model constructed.

### Molecular dynamics

**Figure 7:**
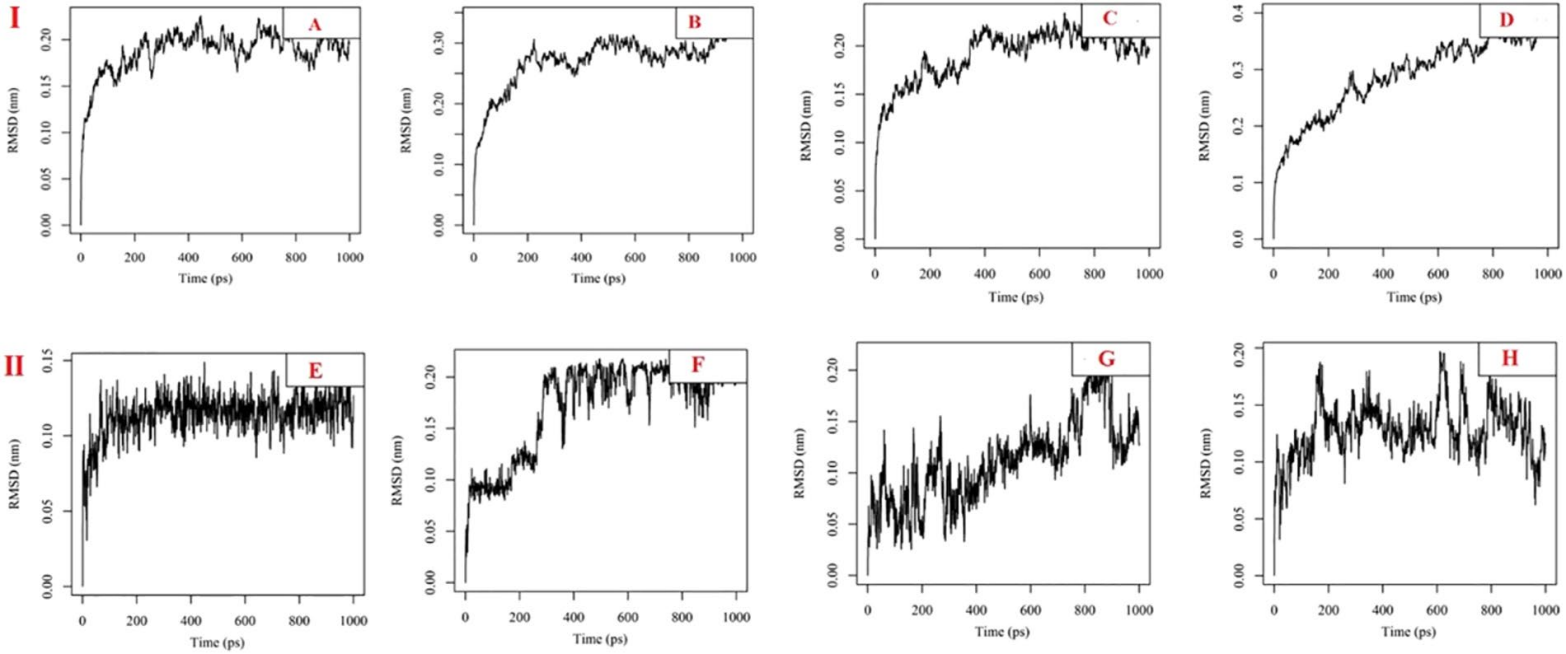
(I) Time dependence of root mean square deviations (RMSDs) of the backbone of protein complexes against the initial structures during 1,000 ps molecular dynamics (MD) simulation.: (**A**) ABA- *C. albicans* complex, (**B**) ABA- *C. parapsilosis* complex, (**C**) ABA- *A. niger* complex, (**D)** ABA- *T. rubrum* complex. (II) Time dependence of root mean square deviations (RMSDs) of the drug candidates against the initial structures during 1,000 ps molecular dynamics (MD) simulation.: (**E)** ABA- *C. albicans* complex, (**F**) ABA- *C. parapsilosis* complex, (**G)** ABA- *A. niger* complex, (**H**) ABA- *T. rubrum* complex.

A molecular dynamic simulation study has been conducted for the four ligand-protein complexes resulted from the docking to obtain more insights into the validity of the docking results achieved and to select phases using the GROMOS force field^20^. The Cα RMSD was calculated and plotted for all of the systems starting from the end of the equilibration phase (Fig. 7I). Through the simulations period, no significant fluctuations were observed in the backbone of the proteins, implying that the binding of ABA at the active sites of the proteins is not only stable and strong but also does not disturb the protein backbone stability.

The ligand positional RMSD of each model was generated and analysed to ensure the binding stability of the ABA in the active site of proteins (Fig. 7II). Both the *C. albicans* and *C. parapsilosis* complexes showed stable and strong binding, while the *A. niger* and *T. rubrum* complexes showed more and continuous fluctuations.

The MD analysis of the proteins and the selected drug candidate’s complex stability were monitored during the trajectory period to determine the stability of hydrogen bonds with the binding site of protein. Hydrogen bond profiles were calculated using the g_h bond utility of GROMACS (Supplementary Material: Fig. SIII). This analysis revealed that the *C. albicans*-abscisic acid complex comprises 5–6 (highest) average H bonds during the simulation period, while the *C. parapsilosis*-abscisic acid, *A. niger*-abscisic acid and *T. rubrum*-abscisic acid complexes showed poor H bond interactions with 2–3 H bonds on average during the trajectory period.

Two different approaches were carried out in order to estimate the binding association between drug candidates and proteins. First, the crude interaction energy was estimated based on the short-range energy of the system. Next, to determine the binding affinity between the protein and drug complexes, the MM-GBSA energy of the complexes was scored^21^. Re-scoring of complexes using g_mmpbsa shows that the selected drug compounds have a different interaction energy of −181.913 kJ/mol, −60.563 kJ/mol, −81.687 kJ/mol, −56.899 kcal/mol in the *C. albicans*-ABA, *C. parapsilosis*-ABA, *A. niger*-ABA, and *T. rubrum*-ABA complexes, respectively. The *C. albicans*-ABA complex displayed a higher binding free energy.

According to the molecular dynamic results, it was strongly predicted that the ABA had a high stability towards blocking the *C. albicans* chorismate mutase active site. It was also predicted that ABA had very good binding to *A. niger*, *C. parapsilosis* and *T. rubrum*.

### *In vitro* antifungal screening for abscisic acid

The previously collected results from virtual screening, molecular docking, and molecular dynamics simulation suggested that ABA had antifungal activity against *C. albicans*, *C. parapsilosis*, *A. niger*, and *T. rubrum* through the inhibition of chorismate mutase enzyme. Therefore, the *in vitro* antifungal activity of ABA against these strains was determined to support the previous *in silico* finding. The *in vitro* antifungal results (Table 4) indicated good antifungal activity of ABA against all the fungal strains, especially *C. parapsilosis* and *T. rubrum*, in comparison with amphotericin B as a positive control.

**Table 4:** The *in vitro* antifungal results for ABA against four strains of fungi; *C. albicans* (RCMB 05036), *C. parapsilosis* (RCMB 05064), *A. niger* (RCMB 02568), and *T. rubrum* (RCMB 010162).

Fungal strain	MIC (µg/mL)	
	(S+)-Abscisic acid	Amphotericin B
*C. albicans*	125	1.95
*C. parapsilosis*	62.5	0.98
*A. niger*	125	1.95
*T.rubrum*	62.5	7.81

The results was expressed in MIC (µg/mL) in comparison with Amphotericin B as a positive control.

### Chorismate mutase assay

Wild-type yeast, *Saccharomyces cerevisiae*, contains a single copy of the chorismate mutase gene, which allows the yeast to convert chorismic acid into prephenic acid (Fig. 1). The latter can be converted into an equivalent concentration of phenylpyruvate through the addition of acid. Thus, the fluctuation in phenylpyruvate or chorismic acid concentrations could be used to indicate yeast chorismate mutase activity. In this assay, yeast chorismate mutase catalysed the conversion of externally added chorismic acid to prephenic acid when favourable conditions were applied. The prephenic acid was converted, non-enzymatically, to phenylpyruvate through the addition of 0.2 N HCl. Phenylpyruvate absorbs UV radiation more effectively in an alkaline medium. Therefore, excess NaOH was added to the reaction medium. HPLC was used to track the amount of chorismic acid utilized as a substrate or/and the amount of phenylpyruvate generated as a product. The established HPLC system enabled the assay of nearly all compounds under investigation: chorismic acid, phenylpyruvate, tyrosine and ABA. However, the tyrosine absorbance and peak resolution were not optimal (Supplementary Materials: Fig. SIV). The system could efficiently separate all of the required metabolites from other yeast metabolites and thus quantify them (Supplementary Materials: Fig. SV).

### Time of reaction

The time needed to reach the maximum product concentration was investigated in the yeast-chorismate-mutase assay system. Chorismic acid was added to the wild-type yeast culture to a final concentration of 1 mM and left at 30 °C. The enzymatic reaction was terminated by the addition of 0.2 N HCl, which simultaneously converted the prephenic acid produced to phenylpyruvate. The acid was added after 5, 10, 20, 45, 60, 90, 120 and 240 minutes. The medium was rendered alkaline using excess NaOH, and the concentration of chorismic acid and phenylpyruvate were identified using HPLC (Supplementary Materials: Fig. SVI). The ideal reaction time to obtain the maximal concentration of the product was 60 minutes (Supplementary Materials: Fig. SVIB). After 240 minutes, there were other products that could be separated by the HPLC system (Supplementary Materials: Fig. SVIA). However, we were not able to identify these products, even by using hyphenated mass spectrometry and library searches. The newly compounds seem to be byproducts of phenylpyruvate, since the amount of phenylpyruvate produced declined.

### Abscisic acid inhibition assay

**Figure 8:**
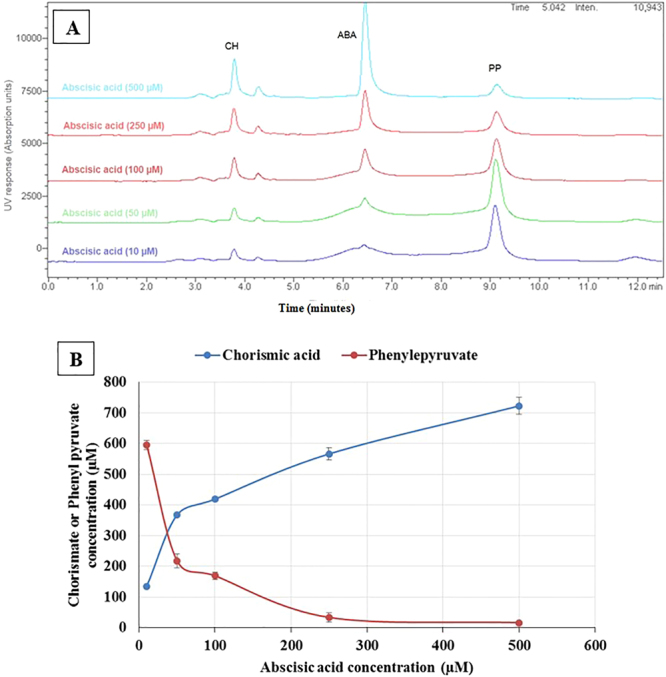
Concentrations of Chorismic acid (CH) and phenyl pyruvate (PP) in response to the addition of Abscisic acid (ABA) in the yeast chorismate mutase assay system. (**A**) HPLC Chromatograms of ABA addition to the yeast-chorismate-mutase-assay system in different concentrations. (**B**) Scatter analysis of chorismic acid and phenylpyruvate concentrations in response to the increase of ABA in the yeast-chorismate-mutase-assay system. The establishment of the yeast-chorismate-mutase-assay system is described in methodology section. Final concentration of CH added was 1000 µM.

To test the inhibitory effect of ABA on chorismate mutase, the established yeast-chorismate-mutase assay system was used. Keeping the concentration of the chorismic acid initially added to 1 mM, various concentrations of ABA were added (500, 250, 100, 50 and 10 µM) to the yeast system. The concentrations of chorismic acid and phenylpyruvate were assessed using the previously established HPLC system, and the results are shown in Fig. 8. The results suggested an inhibitory activity of ABA on chorismate mutase through the inhibition of phenylpyruvate production. The results also indicated that the best concentration of ABA to be used as an inhibitor to yeast chorismate mutase in this system is from 200 to 300 µM.

### Tyrosine inhibition assay

Tyrosine is a known allosteric inhibitor of chorismate mutase. Tyrosine was tested as a chorismate mutase inhibitor in an experiment similar to that of ABA. Various concentrations of tyrosine (500, 250, 100, 50 and 10 µM) were added to the yeast chorismate mutase system. The concentrations of chorismic acid and phenylpyruvate were evaluated (Supplementary Materials: Fig. SVII). The results indicated the inhibitory effect of tyrosine on chorismate mutase through the decline in the phenylpyruvate produced. The optimal concentration for tyrosine to work in this system was 100 µM. This tyrosine experiment can be considered as a validation for the established system of the chorismate mutase assay. Upon comparing the inhibitory activity of tyrosine to that of ABA, it was concluded that tyrosine is 2 to 3 times more potent than ABA.

## Discussion

Fungal infections are highly correlated with increasing rates of mortality, especially among immunocompromised patients^22^. Current treatment choices are limited and hampered by a narrow spectrum, poor bioavailability, toxicity, drug-interactions and by compounds that have fungistatic rather than fungicidal activity^23,24^. Additionally, current medications are ineffective against some fungal infections, such as aspergillosis, and the emergence of multiple drug resistance is also a serious concern^25^. Therefore, the importance of the development of new effective antifungal drugs is imperative.

In this study, the rational drug design approach resulted in repurposing a well-known plant growth-regulating agent, ABA, as a potential fungal chorismate mutase inhibitor, since it might elicit an antifungal effect. Using the previous approach, homology models for chorismate mutase in different fungal strains, *C. albicans*, *C. parapsilosis*, *A. niger*, and *T. rubrum*, were established depending on the crystal structure of the same enzyme in *S. cerevisiae* as a template. The active site for substrate binding was determined through the alignment of chorismate mutase protein sequences of previous fungal strains. The crystal structure of yeast chorismate mutase in complex with *endo*-oxabicyclic transition-state analogue inhibitor^16^ was used to build a pharmacophore model for fungal chorismate mutase inhibitors. The pharmacophore model was used to screen more than 160,000 compounds for their potential anti-chorismate mutase activities, and the top 11 compounds were docked inside the transition-state binding site of the four chorismate mutase homology models of previous fungal strains. Structure similarity searches for the best compounds in previous docking studies led to ABA as a potential fungal chorismate mutase inhibitor. Another molecular docking study was performed for ABA (*S*) and (*R*) isomers against the four homology models and compared to the docking of *endo*-oxabicyclic transition-state analogue inhibitors. The results indicated that the (*S*) isomer is more active and fit to the pharmacophore model. The molecular docking results were supported by the molecular dynamics and *in vitro* antifungal studies. The molecular dynamics studies favoured the interaction between ABA and *C. albicans* homology model of chorismate mutase and gave it the lowest interaction energy. These results not only imply a stable drug-protein interaction but also suggest a long duration of action as well. This property can affect its therapeutic concentration and its dose regimen. The *in vitro* antifungal activity of ABA against the previous fungi strains indicated activity in the microgram range of concentrations that indicates good antifungal activity considering that ABA is not a toxic compound. The established yeast chorismate mutase assay system in this study depended on the release of yeast chorismate mutase after cell disruption, using sonication and in the presence of protease inhibitors. The assay could efficiently separate all the required metabolites. The ideal reaction time needed to reach the product concentration was 60 minutes. The inhibitory concentration of ABA against yeast chorismate mutase ranged from 200 to 300 µM which is a confirmation of the inhibition. Tyrosine, which is a well-known chorismate mutase inhibitor, was compared to ABA and found to be 2 to 3 times more potent than ABA.

ABA is a well-known, naturally occurring sesquiterpene phytohormone that plays fundamental and important roles in plants. ABA regulates plant growth and development, seed and bud dormancy, senescence and abscission, and it exhibits a vital role in plant responses to stresses^26–29^. ABA is produced through the isoprenoid biosynthetic pathway via the degradation of C40 β-carotene derivatives such as zeaxanthin and neoxanthin. The splitting of such products produces xanthoxin, which is the precursor of ABA^30,31^. Although ABA is a major signalling molecule in plants, it was identified in many other organisms such as algae^32^, cyanobacteria^33^ and fungi^34^. ABA was identified in a wide range of lower animals, such as sponges^35^, as well as in higher mammals^36^, including various human tissues and cells^30,37^. Recently, some of the medicinal activities of ABA were identified^38^, including anti-inflammatory^39^, haemopoietic growth factor^40^, antituberculosis^41^, antiatherosclerosis^42^ and anticancer activities^30^. ABA has structural similarities to thiazolidinedione, and consequently it increases the expression of PPAR-γ^43,44^. It is also considered to be an endogenous stimulator of insulin release from human pancreatic islets^45^. These two characteristics contribute to its anti-diabetic activity^30^. ABA exhibits antifungal activity in plants^46^ through the promotion of callose (a β-1,3-glucan polymer) deposition^47^. However, this study suggests another mechanism of ABA as an antifungal through the direct inhibition of the fungal chorismate mutase. ABA is a well-studied molecule that has been shown to be found naturally in the human body^30,37^ and in edible plants. It is a safe and nontoxic with no evidence for carcinogenicity or teratogenicity^48^, which makes it a good target for the pharmaceutical industry. ABA possesses an LD_50_ of more than 5,000 mg/kg in rats^48^, reflecting a wide therapeutic index of the molecule. The compound has only two isomers, which facilitate its synthesis and purification, and it is commercially available.

## Conclusion

ABA was repositioned as a possible anti-chorismate mutase agent with an antifungal activity as a result of a rational drug discovery. This could introduce a new class of antifungal agents, which can contribute to the treatment of life-threatening fungal infections and avoid the severe side effects and toxicity attributed to most currently known antifungal agents. Once again, the rational drug design approach proved to be one of the successful approaches in drug discovery, and it may help the pharmaceutical industry develop and discover more drugs. The effect of the compound on chorismate mutase was confirmed by a chorismate mutase assay in which ABA showed (200 to 300 µM) inhibition, and these results were validated by comparing it to tyrosine which is known to be an allosteric inhibitor of chorismate mutase.

## Methods

### Materials

(+)-Abscisic acid (5 mg, ≥98% (HPLC), m.p. 160 °C) was purchased from Sigma-Aldrich. The compound was tested using its melting point, ^1^H and ^13^C NMR spectroscopy, mass spectrometry and HPLC for structure and purity authentication.

### Sequence similarity search

The pdb search module provided in MOE 2014.09 was used for searching for the best template. Gap start was used as default (−12), Gap extent (−2), E-value cutoff (10) E-value accept (1e-012), Z iterations (100) and Z-cutoff (6).

### Molecular alignment

The molecular alignment was conducted using a Clustal Omega tool provided in the UniProt server (http://www.ebi.ac.uk/Tools/msa/clustalo/). The alignment was conducted between the chorismate mutase FASTA sequence of *Candida albicans* (UniProt ID: Q59TS4), strain (SC5314/ATCC MYA-2876), *Candida parapsilosis* (UniProt ID: G8BD21) strain (CDC 317/ATCC MYA-4646), *Aspergillus niger* (UniProt ID: A2R3Z4) strain (CBS 513.88/FGSC A1513), *Trichophyton rubrum* (UniProt ID: F2SCL7) strain (ATCC MYA-4607/CBS 118892) and *Saccharomyces cerevisiae* (UniProt ID: P32178).

### Homology modelling

The crystal structure of *S. cerevisiae* chorismate mutase (PDB entry code 4CSM) was used as a template with sequence identity 62.82%. *S. cerevisiae* chorismate mutase was used as template chain A, and *C. albicans*, *C. parapsilosis*, *A. niger*, and *T. rubrum* chorismate mutase sequences were used as queries. AMBER99 was used as a force field, and all of the settings were kept as the default in which strain cutoff 1.5 and distance cutoff 1.2 were used.

### Molecular Docking Studies

The molecular docking studies were conducted using MOE 2014.09 and Leadit 2.1.2 software. *Molecular Docking Studies with MOE 2014.09*.

The Molecular Operating Environment (MOE) 2014.09 package license was purchased from Chemical Computing Group Inc., Sherbooke St, Montreal, QC, Canada^49^, and the Leadit 2.1.2 software license was purchased from BioSolveIT GmbH, Germany^50^. All compounds were built and saved as moe. Rigid receptor was used as a docking protocol. Both receptor-solvent were kept as a “receptor”. Triangle matcher was used as a placement method. Two rescoring were computed; rescoring 1 was selected as London dG. Rescoring 2 was selected as affinity. Force field was used as a refinement.

#### Molecular Docking Studies with Leadit 2.1.2

All of the compounds were built and saved as Mol2. The crystal structure of *S. cerevisiae* chorismate mutase in complex with *endo*-oxabicyclic transition state analogue inhibitor was downloaded from a protein databank (pdb code = 4CSM). The protein was loaded into Leadit 2.1.2, and the receptor components were chosen by the selection of chain A as a main chain, which is in complex with the *endo*-oxabicyclic transition state analogue inhibitor. The binding site was defined by choosing the inhibitor as a reference ligand to which all coordinates were computed. Amino acids within a radius of 6.5 Å were selected in the binding site. All chemical ambiguities of residues were left as default. Ligand binding was driven by enthalpy (classic Triangle matching). All default settings were restored for scoring. Intra-ligand clashes were computed by using clash factor = 0.6. Maximum number of solutions per iteration = 200. Maximum of solution per fragmentation = 200. The base placement method was used as a docking strategy.

### Molecular dynamics simulations for protein ligand complexes

Molecular dynamics (MD) simulations were performed using Gromacs 4.5.5^20^. Drug candidates were evaluated within the protein binding sites. The topology file for the selected small molecules were generated using the automated topology builder server PRODRG2^51^ in the framework of GROMOS 53A6 force field^52^. The protein-ligand complexes were then solvated with TIP3P explicit water molecules and placed in the centre of an octahedral box of size 24324 3 24 Å^3^. A minimum 1.0 Å distance was maintained between the protein and the edge of the simulation box, and the periodic boundary condition was used, so that protein can fully immerse with water and rotate freely. Then, the Particle Mesh Ewald (PME) method^53^ was used for the electrostatic energy calculation. It permits the use of the Ewald summation at a computational cost comparable to that of a simple truncation method of 10 Å or less, and the linear constraint solver (LINCS)^54^. An algorithm was used to determine covalent bond constraints. Before minimization, the system was neutralized by the addition of Na^+^ ions. The steepest descent approach (1000 ps) was used for each protein-ligand complex to minimize energy. Further NVT was performed for 20 ps to equilibrate the system with protein and ligand for constant volume, pressure (1 atm) and temperature (300 K). The final MD run was set to 1000 ps for each protein-ligand complex, and trajectories were saved for further analysis using Xmgrace and VMD software^55^. Interaction energy and Gibbs free energy were calculated using Gromacs and g_mmpbsa software, respectively^21^. The interaction energy for the proteins and drug complexes was calculated by estimating the short range Lennard-Jones and short range Coulomb energies using the g_energy analysis tool of Gromacs software. It estimates the stability of proteins and drug candidate complexes. The flowchart that describes all computational steps can be found in (Supplementary Materials: Fig. SVIII).

### *In vitro* antifungal activity

The antifungal assay was conducted as described by Clancy and Nguyen^56^ in comparison with amphotericin B. The minimum inhibitory concentrations were determined at the Regional Center for Mycology and Biotechnology, Al-Azhar University, Cairo, Egypt. The antifungal activity was tested against five fungal strains: *Candida albicans* (RCMB 05036), *Candida parapsilosis* (RCMB 05064), *Aspergillus niger* (RCMB 02568), *Trichophyton rubrum* (RCMB 010162), and *Trichophyton mentagrophytes* (RCMB 010173). An MTT micro dilution assay was used to evaluate the antifungal activity through the determination of the MIC using the serial dilution concentrations of 0.49 µg/mL, 0.98 µg/mL, 1.95 µg/mL, 3.9 µg/mL, 7.81 µg/mL, 15.63 µg/mL, 31.25 µg/mL, 62.5 µg/mL, and 125 µg/mL. The antifungal potency of the drug was calculated as MIC (µg/ml) in comparison with amphotericin B as a positive control.

#### Assay of anti-chorismate mutase activity

Yeast extract (Y1625), glucose (G8270), adenine hemisulphate (A3159), Tris-HCl (T5941), benzamidine HCL (434760), EDTA (E9884), PMSF (78830), chorismic acid (C1259), tyrosine (T3754), phenylpyruvate (P8001) and abscisic acid (A4906) were obtained from Sigma-Aldrich.

#### Yeast cells

Wild type yeast, *Saccharomyces cerevisiae*, was obtained from the Date and Palm Center of Excellence (DPCE), University of King Faisal, Al-Ahsaa, Kingdom of Saudi Arabia. The yeast strain had been previously identified by experts in the Center, and its purity was ensured through sequential streaking and single colony selection.

#### Growth of yeast cells

A single colony of yeast cells was grown in liquid YPAD medium (2% bactopeptone, 1% yeast extract, 2% glucose and 0.04% adenine sulphate). Cultures were grown at 28 °C with shaking at 200 RPM for 48 hours.

#### Production of yeast extracts

Yeast was extracted as described previously with some modifications^57,58^. Briefly, yeast cells were harvested by centrifugation at 4000 × g for 15 minutes and washed with washing buffer (Tris-HCl (50 mM, pH 8.0), benzamidine (1 mM), EDTA (1 mM) and PMSF (1 mM, freshly prepared)) twice. The yeast cells were suspended in this buffer and diluted until the optical density reached an absorbance of 2.0 at 600 nm. Sonication was used to disrupt the yeast cells as described previously^59,60^. Sonication was conducted using a Wise clean sonicator (model WUC-D06H) in 30 °C, 20 kHz (50% of full power). The sonication cycle consisted of 5 minutes of sonication and a 2-minute break, and for 80% yeast cell disruption, 10 cycles were required. Cell disruption was confirmed visually by counting the cells under the microscope in three different fields and comparing with the original cell count before and after sonication. Yeast cell extract was prepared fresh for every experiment.

#### Chorismate mutase assays

The chorismate mutase was assayed as described previously with some modifications.^57,58^ For each experiment, 150 µL of previously made yeast extract was mixed with 100 µL Chorismic acid (10 mM). For experiments using inhibitors, tyrosine (10 mM) or abscisic acid (10 mM), with various volumes according to the experiment, were added to the reaction mixture. The whole components were vortexed and incubated at 30 °C at 150 RPM for various times. The conversion of chorismic acid to prephenic acid was detected through the conversion of the acid produced to the more stable and UV detectable phenylpyruvate by the addition of 250 µL 0.2 N HCl. The reaction was left at room temperature for 10 minutes, and then 500 µL 1 N NaOH was added. The reaction mixture was brought to 1 mL by the addition of Tris-HCl (pH 8) if needed. The reaction mixture was centrifuged at 12,000 RPM for 10 minutes, and the supernatant was subjected to HPLC analysis.

#### HPLC analysis

HPLC analysis, including chorismic acid, phenylpyruvate, tyrosine and abscisic acid separation and quantification, was performed using a Shimadzu Prominence HPLC system equipped with a CBM-20A controller, LC-20A solvent unit, SIL-20A auto-sampler, CTO-20A column oven and SPD-20A UV-VIS detector. The compounds were separated using a Luna-C_18_ (L1, Phenomenex, 150 mm × 4.6 mm × 5 μm). Mobile phases were a combination of 0.1% trifluoroacetic acid in water: acetonitrile: methanol (50:40:10). The flow rate was adjusted to 0.5 mL/minute, and the UV detector was adjusted to 320 nm. Calibration curves for chorismic acid, phenylpyruvate, tyrosine and abscisic acid were performed using standards.

#### Identification of Phenylpyruvate

To identify phenylpyurate, HPLC analysis was performed using an Agilent LC-MS platform (1200 series) comprising a pump system, photodiode array detector and a Quadrupole mass spectrometer (model 6120, detecting between 150 and 600 m/z units, with electrospray positive ionization (ESI^+^)). The same chromatographic conditions described above were applied. The phenylpyruvate produced by the yeast cultures was identified through the recognition of the new peak produced after the addition of chorismic acid to the cultures. The mass chromatogram of the new peak was compared to mass spectral data in Wiley Registry of Mass Spectral Data 10th Edition (April 2013) and NIST 11 Mass Spectral Library (NIST11/2011/EPA/NIH). Spiking experiments with authentic phenylpyruvate were also used to confirm the identity of the newly produced peak. The flowchart that describes the chorismate mutase *in vitro* assay can be found in (Supplementary Materials: Fig. SVIX).

## Electronic supplementary material

supplementary material
