# Supplementary material for: Rational Discovery of (+) (S) Abscisic Acid as a Potential Antifungal Agent: a Repurposing Approach
Source: Sci Rep. 2018 Jun 4;8:8565. doi: 10.1038/s41598-018-26998-x (PMC5986790; doi:10.1038/s41598-018-26998-x)
Supplement: Supplementary file 1 — supplementary material [file 41598_2018_26998_MOESM1_ESM.pdf]

Rational Discovery of (+) (*S*) Abscisic Acid as a Potential Antifungal Agent: a Repurposing Approach

Mohammed A. Khedr [1, 2], * Alberto Massarotti [3], and Maged E. Mohamed [1,4]

[1] College of Clinical Pharmacy, King Faisal University, P.O. 380, Al-Hasaa 31982, Kingdom of Saudi Arabia

[2] Department of Pharmaceutical Chemistry, Faculty of Pharmacy, Helwan University, Ein Helwan, Cairo, 11795, Egypt

[3] Dipartimento di Scienze del Farmaco, Università del Piemonte Orientale "A. Avogadro" Largo Donegani 2, 28100 Novara, Italy

[4] Department of Pharmacognosy, Faculty of Pharmacy, University of Zagazig, Zagazig, 44519, Egypt

Corresponding Author:

Mohammed A. Khedr

Associate professsor of medicinal chemistry

E-mail: mmohammed@kfu.edu.sa; mohammed_abdou0@yahoo.com

Tel.: +966545045648;

Fax: +966-3-5817174-3

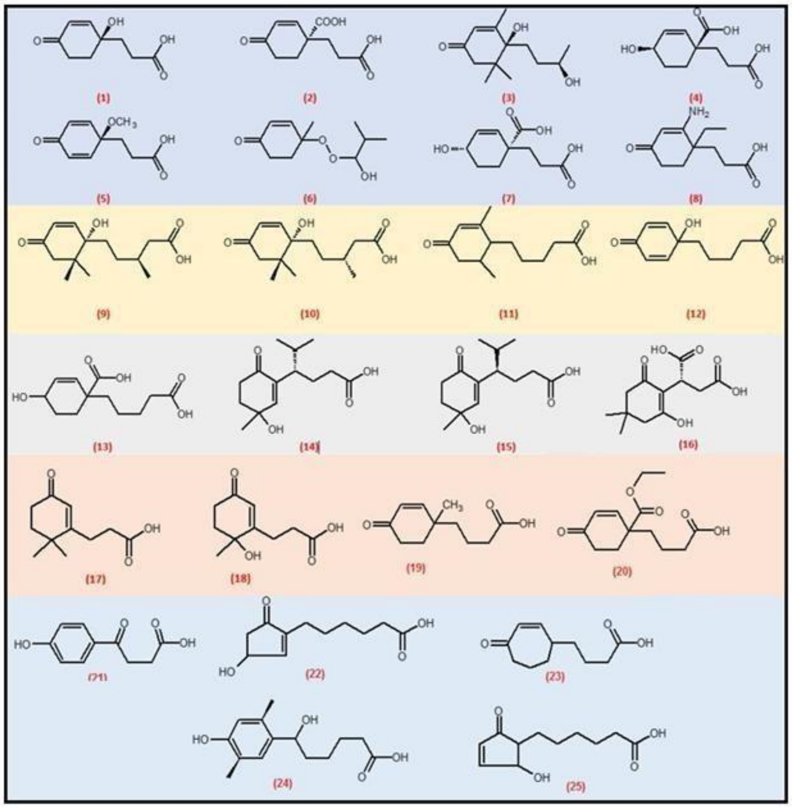

Figure SI. Compounds retrieved after screening of a number of databases such as the MOE 2014.09, and the ZINC databases for the established pharmacophore model of fungal chorismate mutase inhibitors. The shown 25 compounds were selected as top-ranked hits in the screening with RMSD < 1.00 and retaining all pharmacophore features.

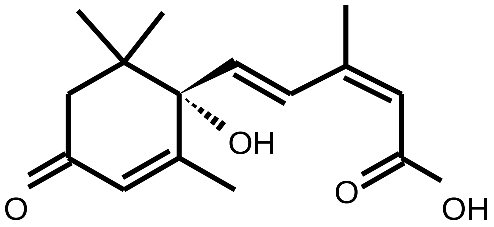

(+) (*S*) Abscisic Acid

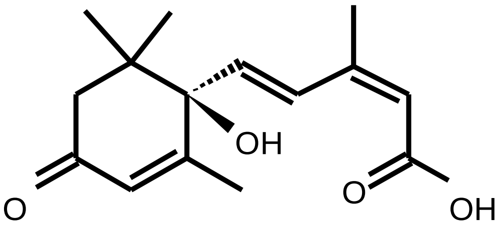

(-) (*R*) Abscisic Acid

Figure SII. Different stereo-configuration of Abscisic acid.

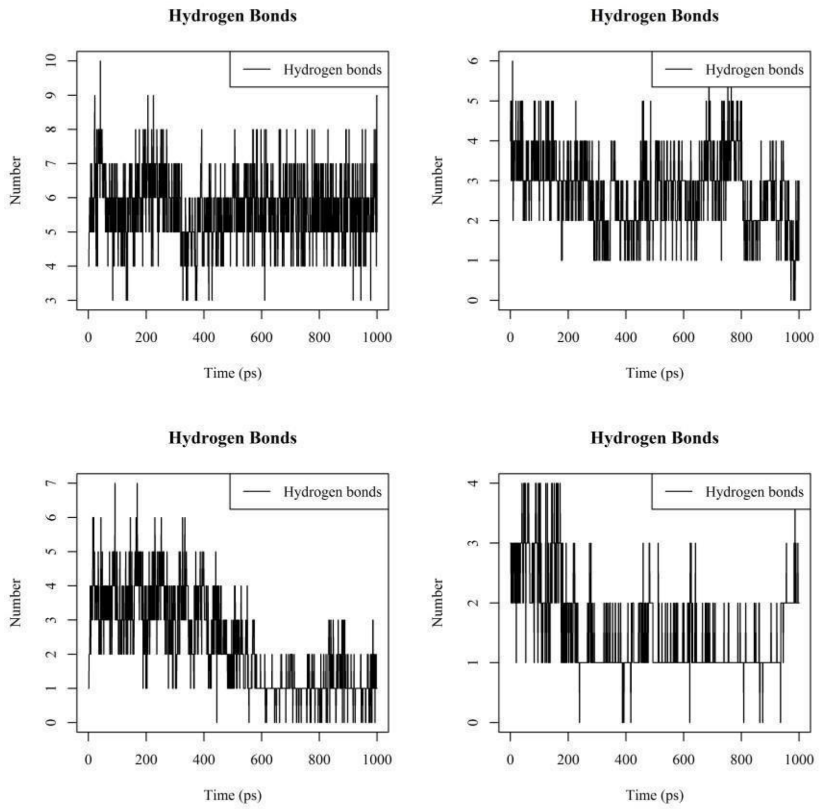

Figure SIII. Hydrogen bonds count formed between the ligand and receptor molecules across the production phase trajectory. The plots represent the systems of structure complexes with the original PDB ligand coordinates and docked hits coordinates in the order: **A)** ABA- *C. albicans* complex, **B)** ABA- *C. parapsilosis* complex, **C)** ABA- *A. niger* complex, **D)** ABA- *T. rubrum* complex.

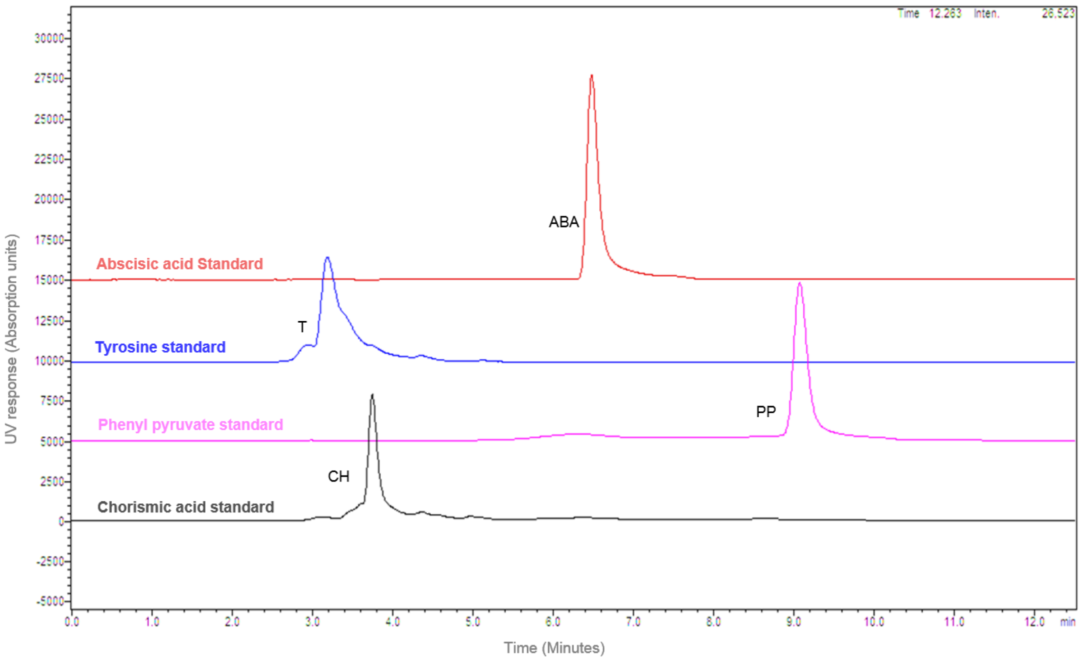

Figure SIV: HPLC for simultaneous detection of Chorismic acid (CH), Phenyl pyruvate (PP), Tyrosine (T) ad Abscisic acid (ABA). The HPLC system and detection are described in the material and method section.

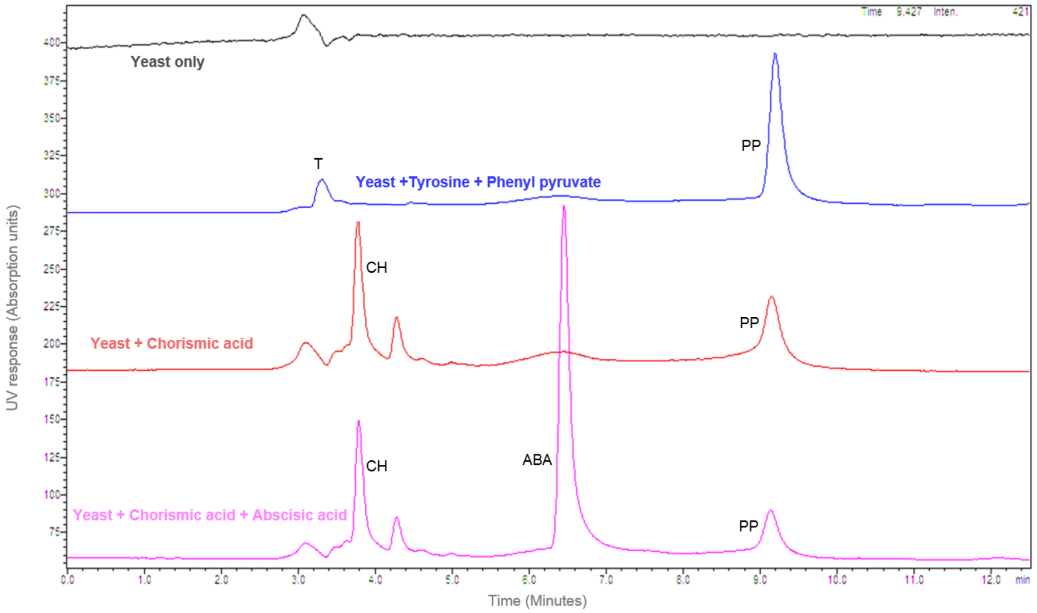

Figure SV: The yeast Chorismate mutase assay system. clear separation and identification of metabolites peaks using HPLC. Chorismic acid (CH), Phenyl pyruvate (PP), Tyrosine (T), Abscisic acid (ABA). The yeast Chorismate mutase assay system is described in the material and method section.

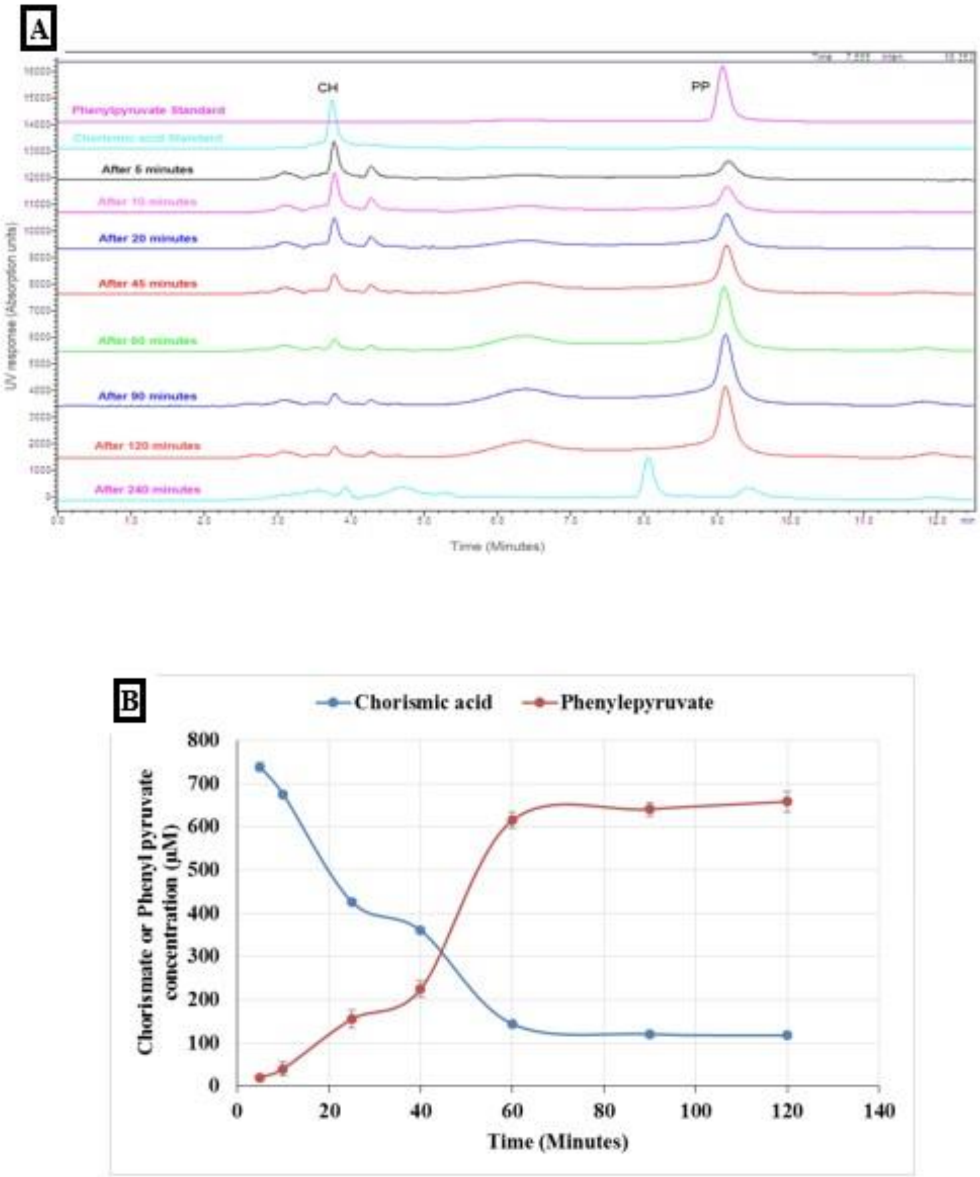

Figure SVI: Concentrations of Chorismic acid (CH) and phenyl pyruvate (PP) in response to reaction time in the yeast chorismate mutase assay system. **A**: HPLC Chromatograms of the yeast-chorismate-mutaseassay system in different time reaction intervals. **B**: Scatter analysis of chorismic acid and phenyl pyruvate concentrations in response to the increase of time (in minutes) in the yeast-chorismate-mutase-assay system. The establishment of the yeast-chorismate-mutase-assay system is described in methodology section. Final concentration of CH added was 1000 µM.

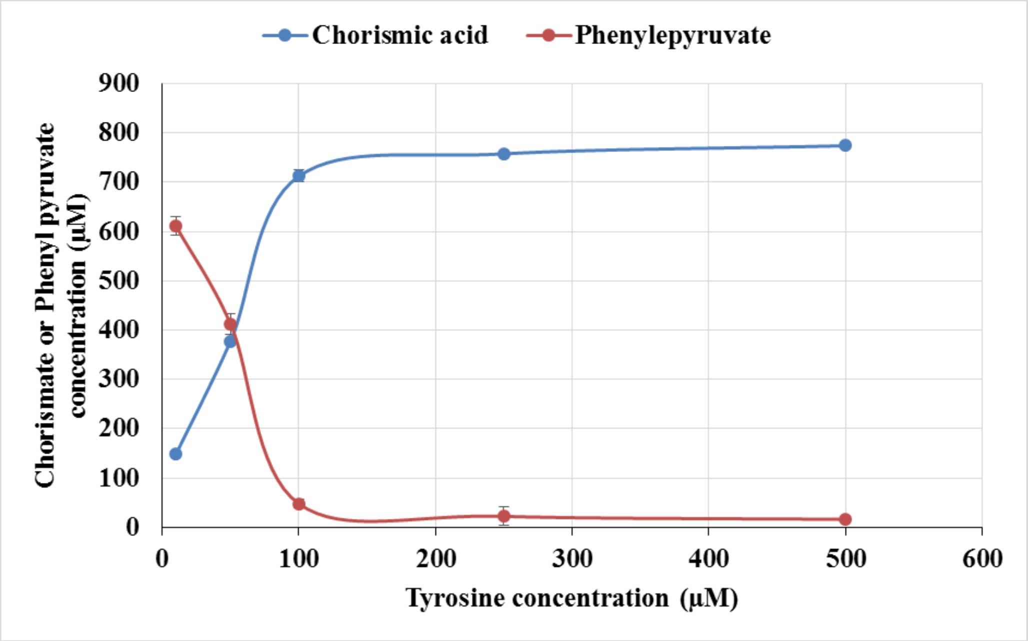

Figure SVII: Concentrations of Chorismic acid (CH) and phenyl pyruvate (PP) in response to the addition of Tyrosine in the yeast chorismate mutase assay system. **A**: HPLC Chromatograms of Tyrosine addition to the yeast-chorismate-mutase-assay system in different concentrations. **B**: Scatter analysis of chorismic acid and phenyl pyruvate concentrations in response to the increase of Tyrosine concentration in the yeastchorismate-mutase-assay system. The establishment of the yeast-chorismate-mutase-assay system is described in methodology section. Final concentration of CH added was 1000 µM.

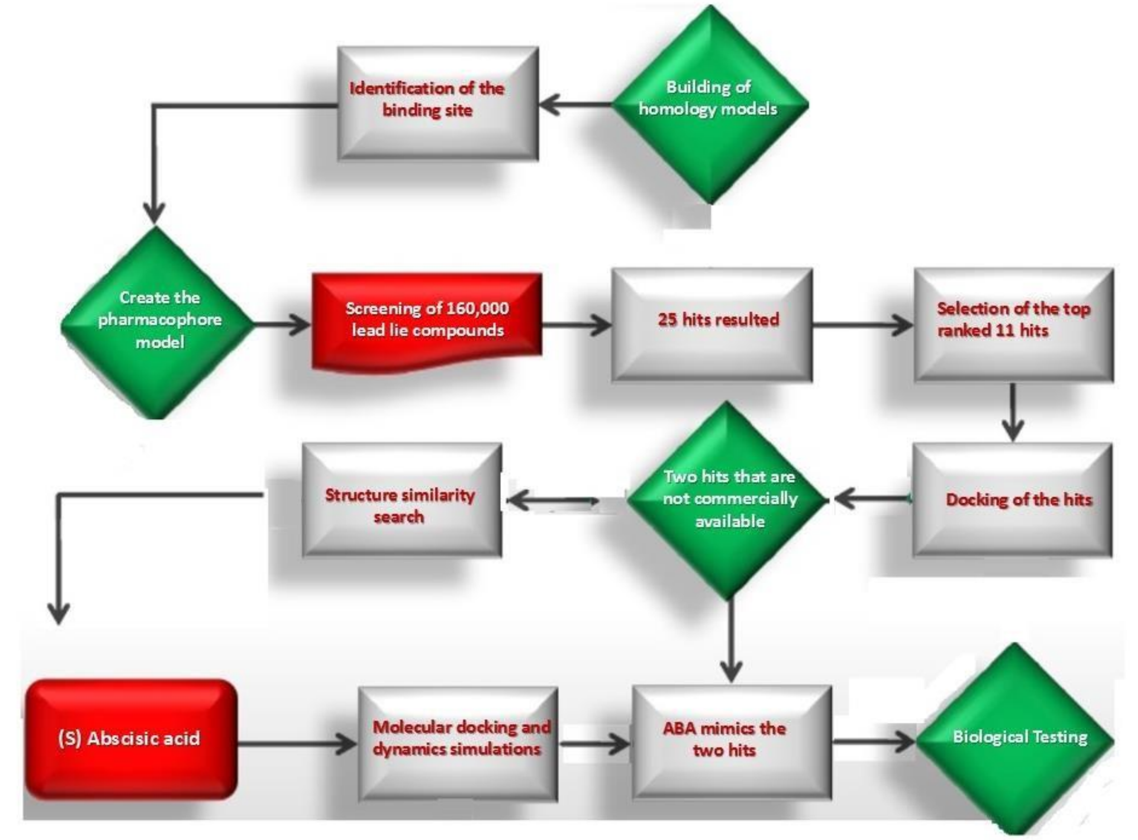

Figure SVIII: Flowchart showing the steps of the virtual screening process until the discovery of ABA as a hit that is matched with all pharmacophoric features. The last step includes the biological testing that is fully described in the following Flowchart 2

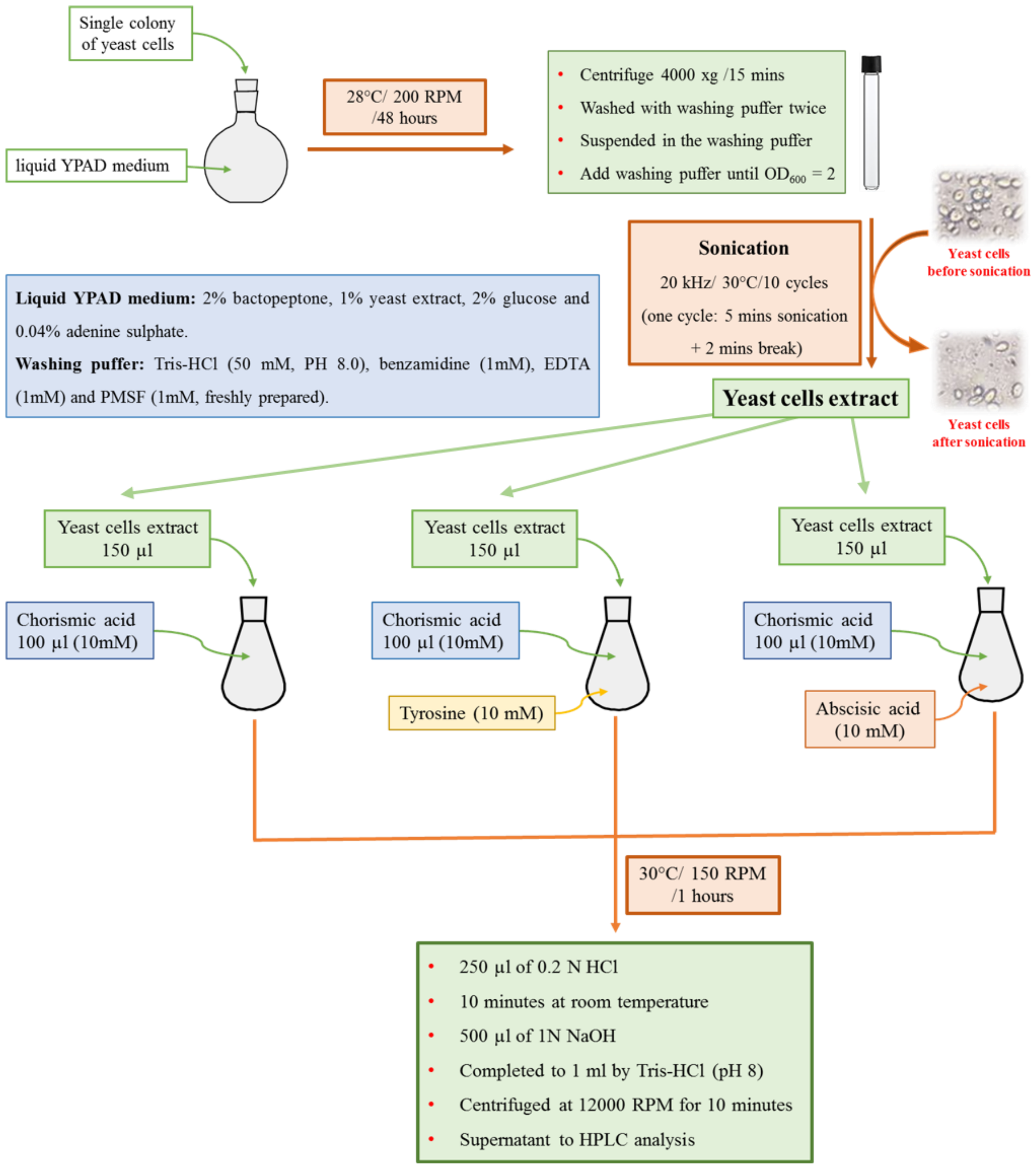

Figure SIX: Flowchart showing the steps of the yeast chorismate mutase in vitro assay. Yeast cells magnification power 40X.
